# Neo-Adjuvant Chemotherapy Reduces, and Surgery Increases Immunosuppression in First-Line Treatment for Ovarian Cancer

**DOI:** 10.3390/cancers13235899

**Published:** 2021-11-24

**Authors:** Christine De Bruyn, Jolien Ceusters, Chiara Landolfo, Thaïs Baert, Gitte Thirion, Sandra Claes, Ann Vankerckhoven, Roxanne Wouters, Dominique Schols, Dirk Timmerman, Ignace Vergote, An Coosemans

**Affiliations:** 1 Laboratory of Tumor Immunology and Immunotherapy, ImmunOvar Research Group, Department of Oncology, Leuven Cancer Institute, Katholieke Universiteit Leuven, 3000 Leuven, Belgium; christine.debruyn@uzleuven.be (C.D.B.); jolien.ceusters@kuleuven.be (J.C.); chiara.landolfo@policlinicogemelli.it (C.L.); thais.baert@uzleuven.be (T.B.); gitte.thirion@uzleuven.be (G.T.); ann.vankerckhoven@kuleuven.be (A.V.); roxanne.wouters@kuleuven.be (R.W.); 2 Department of Obstetrics and Gynecology, University Hospital Antwerp, 2650 Edegem, Belgium; 3 Department of Obstetrics and Gynecology, Leuven Cancer Institute, University Hospitals Leuven, 3000 Leuven, Belgium; dirk.timmerman@uzleuven.be (D.T.); ignace.vergote@uzleuven.be (I.V.); 4 Dipartimento Scienze della Salute della Donna, del Bambino e di Sanità Pubblica, Fondazione Policlinico Universitario Agostino Gemelli, Istituto di Ricovero e Cura a Carattere Scientifico (IRCCS), 00168 Rome, Italy; 5 Department of Development and Regeneration, Katholieke Universiteit Leuven, 3000 Leuven, Belgium; 6 Queen Charlotte’s and Chelsea Hospital, Imperial College, London W12 0HS, UK; 7 Department of Gynecology and Gynecologic Oncology, Kliniken Essen Mitte, 45136 Essen, Germany; 8 Laboratory of Virology and Chemotherapy (Rega Institute), Department of Microbiology, Immunology and Transplantation, Katholieke Universiteit Leuven, 3000 Leuven, Belgium; Sandra.Claes@kuleuven.be (S.C.); dominique.schols@kuleuven.be (D.S.); 9 Oncoinvent AS, 0484 Oslo, Norway; 10 Department of Oncology, Gynaecological Oncology, Katholieke Universiteit Leuven, 3000 Leuven, Belgium

**Keywords:** ovarian cancer, chemotherapy, immunosuppression, debulking surgery, neo-adjuvant chemotherapy

## Abstract

**Simple Summary:**

The immune system plays an important role in the development and progression of cancer. The current treatments for ovarian cancer (surgery and chemotherapy) create changes in the immune system, but it is not clear how. Nevertheless, if immunotherapy is associated on top of this, then it seems crucial to understand what is changing in the current state of the art. In this study, we measured immune-related proteins in the serum of ovarian cancer patients throughout their treatment. We discovered that carboplatin–paclitaxel as a chemotherapeutic treatment reduces immunosuppression and promotes immunostimulation, meaning that the immune system be-comes less hostile and more in favour of the patient. Therefore, chemotherapy seems to induce a temporary window of opportunity to insert immunotherapy during the current treatment of ovarian cancer patients.

**Abstract:**

In monotherapy, immunotherapy has a poor success rate in ovarian cancer. Upgrading to a successful combinatorial immunotherapy treatment implies knowledge of the immune changes that are induced by chemotherapy and surgery. Methodology: Patients with a new ovarian cancer diagnosis underwent longitudinal blood samples at different time points during primary treatment. Results.: Ninety patients were included in the study (33% primary debulking surgery (PDS) with adjuvant chemotherapy (ACT), 61% neo-adjuvant chemotherapy (NACT) with interval debulking surgery (IDS), and 6% debulking surgery only). Reductions in immunosuppression were observed after NACT, but surgery reverted this effect. The immune-related proteins showed a pronounced decrease in immune stimulation and immunosuppression when primary treatment was completed. NACT with IDS leads to a transient amelioration of the immune microenvironment compared to PDS with ACT. Conclusion: The implementation of immunotherapy in the primary treatment schedule of ovarian cancer cannot be induced blindly. Carboplatin–paclitaxel seems to ameliorate the hostile immune microenvironment in ovarian cancer, which is less pronounced at the end of primary treatment. This prospective study during primary therapy for ovarian cancer that also looks at the evolution of immune-related proteins provides us with an insight into the temporary windows of opportunity in which to introduce immunotherapy during primary treatment.

## 1. Introduction

Ovarian cancer is the 8th most commonly diagnosed cancer among females and the 8th most frequent cause of female cancer-related deaths worldwide [1]. Most ovarian cancer patients are diagnosed in an advanced stage (FIGO stage III-IV) and have a significantly lower 5-year survival rate compared to stage I ovarian cancer (93.3 (95%CI 91.6–95.0%) versus 26.9% (95%CI 25.3–28.6%)) [2]. More than 90% of ovarian cancers are epithelial in origin, with 70% of them being high-grade serous ovarian cancer [3]. Primary therapy consists of cytoreductive debulking surgery with platin-based chemotherapy [4]. Progress has been made in the last few years with the addition of Bevacizumab^®^ (VEGF (vascular endothelial growth factor) monoclonal antibody) [5,6,7] and PARP (poly-adenosine diphosphate-ribose polymerase) inhibitors [8,9,10,11] as maintenance therapy.

Therapy response is evaluated clinically by regular radiological evaluations and serial measurements of carbohydrate antigen 125 (CA125). At diagnosis, CA125 is raised in 85% of advanced disease cases. Although it remains the most relevant protein that can be used to discriminate between benign and malignant disease at diagnosis [12], it is still a non-specific protein for ovarian cancer. Increased CA125 levels are also observed in benign diseases (e.g., endometriosis, pelvic inflammatory disease, pregnancy, and ovarian cysts) and non-gynaecological malignancies (e.g., breast, lung, colon, and pancreatic cancer) [13,14].

The immune system plays an important role in the development and progression of cancer [15]. For ovarian cancer, the emphasis has been on the adaptive immune system for a long time [16,17]. However, the importance of the innate immune system has recently been demonstrated [18,19,20]. So far, immunotherapeutic strategies have been focusing nearly solely on the manipulation of the adaptive immune system. Immune checkpoint inhibition has shown a poor response in monotherapy [21,22], but combinatorial immunotherapy trials have also been unable to meet the high expectations that are placed on these trials (e.g., JAVELIN ovarian 100 and 200 trial, IMagyn050/GOG 3015/ENGOT-OV39 trial) [23,24,25]. Currently, combination treatments are failing, which is most likely because knowledge on how to combine treatments is lacking. Both chemotherapy, surgery, and immunotherapy will alter the immune microenvironment, but the extent to which this is possible has yet to be explored [26]. However, this knowledge is mandatory if we want to combine treatments in an effective way.

In this manuscript, we highlight the changes in immune related proteins throughout the primary state-of-the-art treatment for ovarian cancer. These proteins have also recently been proven to be relevant in the discrimination between benign and malignant tumours [12]. This systematic analysis, which has been conducted throughout the treatment process, highlights for the first time the most optimal and transient moment to combine immunotherapy in first-line treatment. 

## 2. Results

### 2.1. Patient Characteristics

Ninety patients were included in the study. The median age of the study population at diagnosis was 64 years (range 26–88 years). Eighty-six percent of the study population had an advanced stage of disease (FIGO stage III or IV). High grade serous ovarian cancer was the most frequent histology (72%). In total, 18% of the patients did not undergo surgery. Chemotherapy was administered in 85 patients, and in 61%, chemotherapy was administered in an upfront neoadjuvant setting. Five patients (6%) received no chemotherapy based on their FIGO stage and histology. Patients who did not receive chemotherapy were excluded from further analyses. Relapse occurred in 60% (54/90) of the patients within a median of 22 months (95%CI of 17–36 months). The median follow-up was 35.5 months. The progression-free survival at 3 years was 24% (22/90), with a disease-specific survival of 64% (58/90). Further patient characteristics are presented in Table 1.

### 2.2. Serum Characteristics

Originally, 23 proteins were included for analysis in the study. Due to a small percentage of observed protein concentrations (missing values or out-of-ranges values), 7 proteins were excluded from the analysis (IL-12, IL-17, MCP-1, MMP-12, MMP-13, TGF-β and Galectin-3). Hence, 16 proteins were used in the final analyses. More information can be found in Figure S1 in the Supplementary Materials. The immune related proteins were subdivided into three categories: immune stimulatory factors (CXCL 9, CXCL 10, CXCL 12, CCL 5), immune inhibitory factors (CCL11, CCL24, MMP1-7-8-9, arginase, VEGF, IL-10, SAA, and osteopontin), or dual function (CCL22).

### 2.3. Neoadjuvant Carboplatin Reduces Immunosuppression in Ovarian Cancer Patients

**Figure 1:**
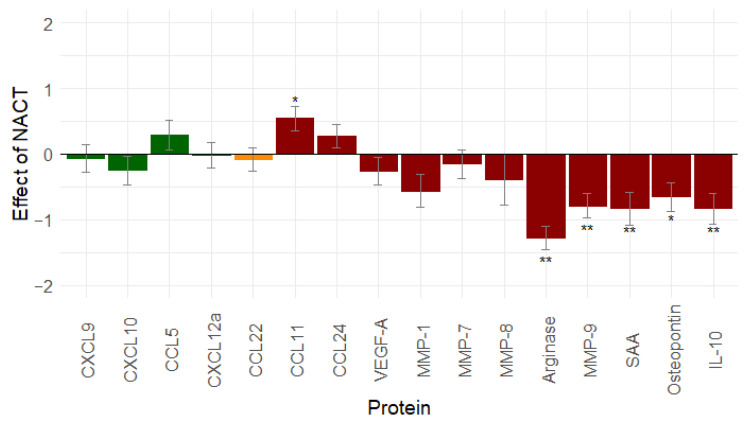
Effect of neo-adjuvant chemotherapy (*n* = 55) on immune-related proteins: platin-based neo-adjuvant chemotherapy mainly decreases immunosuppression. Proteins in green are immune stimulatory factors, proteins in red are immune inhibitory factors, proteins in orange have a dual function. *: 0.01 ≤ *p*-value < 0.05; **: *p*-value < 0.01. Abbreviations: CCL, C-C motif chemokine ligand; CXCL, C-X-C motif chemokine ligand; IL-10, interleukin-10; MMP, matrix metallopeptidase; NACT, neo-adjuvant chemotherapy; SAA, serum amyloid A; vascular endothelial growth factor (VEGF).

To demonstrate the effect of neoadjuvant chemotherapy (NACT) on the immune-related proteins, the serum samples that were taken at diagnosis were compared with serum samples after platin-based NACT (*n* = 55). Results are shown in Figure 1.

Thirteen proteins decreased after the NACT treatment. Only CCL11, CCL24, and CCL5 increased. A significant change was found for six proteins: CCL11 (*p* = 0.013), Arginase (*p* < 0.001), MMP-9 (*p* < 0.001), SAA (*p* = 0.001), osteopontin (*p* = 0.011), and IL-10 (*p* = 0.001). Based on this result, NACT mainly seems to reduce immunosuppression.

Of note, if the NACT patients who only received carboplatin–paclitaxel (*n* = 10) are compared to those receiving bevacizumab (Avastin^®^) (*n* = 32), then the conclusions are comparable. However, the decrease in the arginase, SAA, and IL-10 concentration compared to the values at diagnosis becomes significant in contrast to chemotherapy alone. Results are displayed in the Supplementary Materials (Table S1).

To evaluate if the effects of carboplatin–paclitaxel on the immune system could be influenced by the tumour load, we looked at patients in the adjuvant chemotherapy (ACT) group with R0 resection (*n* = 30), comparing serum samples after primary debulking surgery with serum samples in the middle of adjuvant chemotherapy. The results were similar, with a predominant decrease in immunosuppression (Figure S2). We can therefore conclude that it is the presence of carboplatin–paclitaxel that is able to reduce immunosuppression. 

### 2.4. Debulking Surgery Creates an Unfavourable Immune Environment

The effect of surgery was evaluated by comparing serum samples after NACT with serum samples after interval debulking surgery (IDS) in the NACT group and serum samples at diagnosis, with serum samples after primary debulking surgery (PDS) being taken in the ACT group. Only patients with a complete resection of their tumour load (R0 resection: *n* = 66) were included in the analysis.

**Figure 2:**
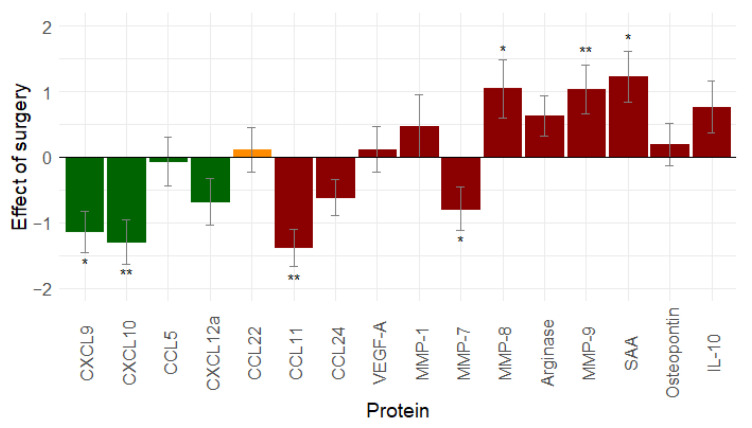
Effect of debulking surgery (*n* = 66) on immune related proteins: debulking surgery tends to increase immunosuppression and decrease immunostimulation (similar results are seen when leaving out stage I ovarian cancer patients, see Figure S3). Proteins in green are immune stimulatory factors, proteins in red are immune inhibitory factors, and proteins in orange have a dual function. *: 0.01 ≤ *p*-value < 0.05; **: *p*-value < 0.01. Abbreviations: CCL, C-C motif chemokine ligand; CXCL, C-X-C motif chemokine ligand; IL-10, interleukin-10; MMP, matrix metallopeptidase; SAA, serum amyloid A; vascular endothelial growth factor (VEGF).

Seven proteins decreased after surgery, and nine proteins increased. A significant change was found for seven proteins: CCL11 (*p* < 0.001), CXCL10 (*p* = 0.001), CXCL9 (*p* = 0.012), MMP-7 (*p* = 0.040), MMP-8 (*p* = 0.049), MMP-9 (*p* = 0.002), and SAA (*p* = 0.018) (Figure 2). Debulking surgery seems to result in a decrease in immune stimulation and an increase in immunosuppression.

To evaluate the effect of platin-based chemotherapy prior to surgery, samples from the NACT group (*n* = 36) and the ACT group (*n* = 30) were compared (Figure S4). The trend in the decreasing/increasing immunostimulation/immunosuppression was the same in both groups. However, in the NACT group, a significant change was observed in CCL11 (*p* < 0.001), MMP-7 (*p* = 0.029), MMP-9 (*p* = 0.022), and SAA (*p* = 0.004). A significant change was only observed for two proteins in the ACT group: CXCL9 (*p* = 0.014) and CXCL10 (*p* = 0.003).

### 2.5. Adjuvant Chemotherapy Leads to Mixed Immune Changes

To evaluate the effect of adjuvant chemotherapy, serum samples after IDS and at the end of treatment in the NACT group were compared. In the ACT group, the serum samples in the middle of chemotherapy were compared with serum samples at the end of treatment. Only patients with the complete tumour resection during surgery (R0 resection: *n* = 66) were included in these analyses.

**Figure 3:**
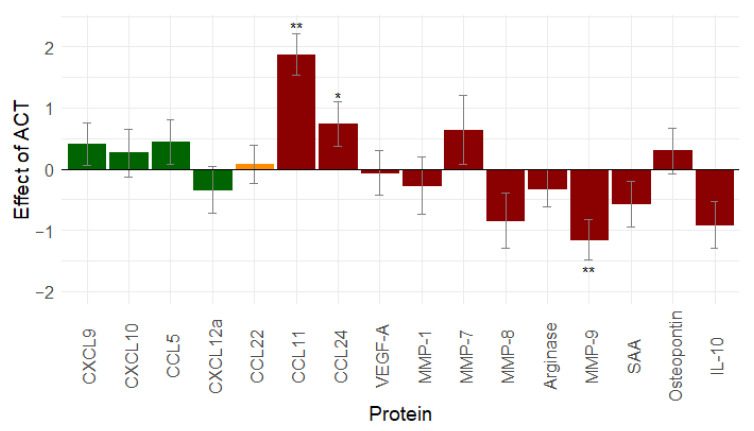
Effect of adjuvant chemotherapy (*n* = 66) on immune related proteins: adjuvant chemotherapy shows non-significant increase in immunostimulation and a mixed effect on immunosuppression (similar results are seen when leaving out stage I ovarian cancer patients, see Figure S5). Proteins in green are immune stimulatory factors, proteins in red are immune inhibitory factors, and proteins in orange have a dual function. *: 0.01 ≤ *p*-value < 0.05; **: *p*-value < 0.01. Abbreviations: CCL, C-C motif chemokine ligand; CXCL, C-X-C motif chemokine ligand; IL-10, interleukin-10; MMP, matrix metallopeptidase; ACT, adjuvant chemotherapy; SAA, serum amyloid A; vascular endothelial growth factor (VEGF).

Eight proteins decreased after ACT treatment, and eight proteins increased. A significant change was observed for three proteins: CCL11 (*p* < 0.001), CCL24 (*p* = 0.031), and MMP-9 (*p* < 0.001) (Figure 3). From our analyses, it seems that adjuvant chemotherapy can (partially) revert the effect of the debulking surgery through a non-significant increase in immune stimulation and through a rather mixed effect on immunosuppression.

### 2.6. Complete Primary Treatment Results in a Less Hostile Immune Environment Compared to the Situation at Diagnosis

**Figure 4:**
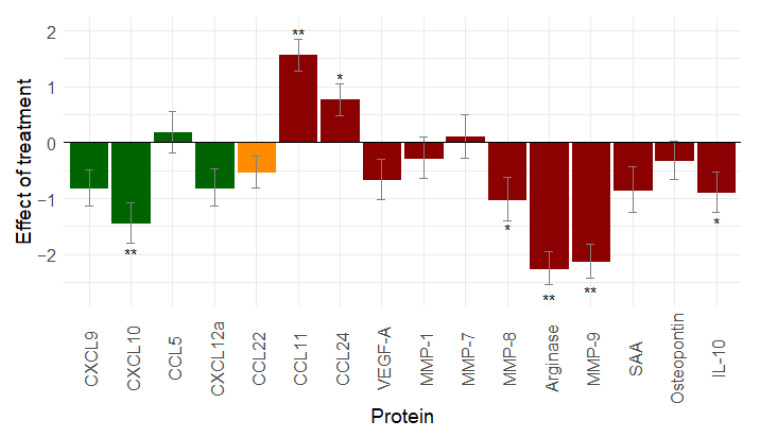
Effect of total primary treatment (*n* = 66) on immune related proteins: primary treatment of ovarian cancer gives a pronounced decrease in immunostimulation and immunosuppression. (Similar results are seen when leaving out stage I ovarian cancer patients, see Figure S6.) Proteins in green are immune stimulatory factors, proteins in red are immune inhibitory factors, and proteins in orange have dual function. *: 0.01 ≤ *p*-value < 0.05; **: *p*-value < 0.01. Abbreviations: CCL, C-C motif chemokine ligand; CXCL, C-X-C motif chemokine ligand; IL-10, interleukin-10; MMP, matrix metallopeptidase; SAA, serum amyloid A; vascular endothelial growth factor (VEGF).

To evaluate the effect of the complete primary treatment, we compared the serum samples that were taken at diagnosis with the serum samples that were taken at the end of the treatment. Only patients with complete tumour resection during surgery (R0 resection: *n* = 66) were included in the analysis. Twelve proteins decreased after the primary treatment. Only CCL11, CCL24, MMP-7, and CCL5 increased. A significant change was observed for seven proteins: CCL11 (*p* < 0.001), CCL24 (*p* = 0.02), CXCL10 (*p* < 0.001), MMP-8 (*p* = 0.039), arginase (*p* < 0.001), MMP-9 (*p* < 0.001), and IL-10 (*p* = 0.038) (Figure 4).

The overall effect of the primary treatment on immune-related proteins is a decrease in immune stimulation and immunosuppression, rendering the environment less hostile after treatment than it was at diagnosis.

### 2.7. Sequence in Surgery and Chemotherapy Transiently Influences the Immune Environment

To evaluate if there is an immunological difference when the order of chemotherapy and surgery is altered (i.e., first chemotherapy, then surgery (NACT group: *n* = 36) versus surgery first followed by chemotherapy (ACT group: *n* = 30)), we compared the serum samples that were taken at diagnosis and after IDS with the serum samples that were taken at diagnosis and in the middle of adjuvant chemotherapy. Interestingly, we indeed observed significant differences between the groups concerning the concentrations of CCL5 (*p* = 0.0013), CCL22 (*p* = 0.0004), MMP-7 (*p* = 0.0160) and IL-10 (*p* = 0.0002) (Table 2). Overall, we observed a more pronounced decrease in the immunosuppressive proteins in the NACT group compared to in the ACT group.

### 2.8. Progression-Free Survival Analyses

The levels in the immune-related proteins at different time points in the treatment schedule (i.e., after NACT, ACT, surgery, and total primary treatment) were related to PFS by calculating the hazard ratios (HR). None of them were significant (results are not shown); therefore, it is impossible to draw any conclusions at this point. A larger prospective study will be necessary. OS data are immature, with only 34 events at the time of analyses. 

## 3. Discussion

To our knowledge, this study is the first prospective cohort study trial on the evolution of immune related proteins during first-line treatment in ovarian cancer. Platin-based chemotherapy does alter the immune system in a favourable way by reducing immune suppression. Surgery reverses this effect by rendering the environment more hostile. ACT can reverse the negative effect of surgery on the immune system. Importantly, the order in which chemotherapy and surgery are applied to a patient alters the immune environment differently, with a transient benefit for NACT. Complete first-line treatment in ovarian cancer renders the immune microenvironment less hostile, which is mainly the result of a decrease in immunosuppression. This prospective cohort study confirms our previously published retrospective results [29].

Until now, only a small number of publications have focused on the effects of chemotherapy on the immune system in ovarian cancer [30], most of them being retrospective and focusing on tumour biopsy samples. However, studies using tumour biopsies have the potential shortcoming that the immune microenvironment in ovarian cancer is highly susceptible to intrapatient spatiotemporal variation [31,32,33]. Systemic immune profiling with peripheral blood samples might be more representative [34]. To our knowledge, only two studies have been published on systemic immune profiling. In 2010, Wu et al. observed a significant increase of CD8+ T cells and a decrease in regulatory T cells (Treg) at day 12–14 after one cycle of adjuvant carboplatin–paclitaxel in 13 patients with advanced primary epithelial ovarian cancer [35]. A longitudinal study in nine patients by Colleman et al. in 2005 showed a positive effect of adjuvant platin-based chemotherapy on the function of CD8+ T cells in ovarian cancer [36]. In other cancers, for example lung cancer, the results are less uniform [37,38]. Multiplex protein biomarker data pre- and post-chemotherapy is as good as not existing in the literature.

Surgery induces a immunosuppressive state to support wound healing and postoperative pain [39]. Altered cytokine levels (decreases in IL-2, IL-12, IFN- γ and increases in IL-6, Il-8, TNF-α) and the release of growth factors (VEGF and TGF-β) generate an increase in Treg, MDSC (myeloid derived suppressor cells), and M2 macrophages and a decrease in CD8+ T cells. Postoperative complications (e.g., sepsis, blood loss, hypothermia, …) were shown to aggravate the immunosuppressed state [39]. Specifically for ovarian cancer, primary debulking surgery decreases Treg cells in the peripheral blood at day 1 postoperatively, with an increase at day 7 postoperatively [40]. Suboptimal debulking surgery worsens the immunosuppressive state due to increased levels of TGF-β [41].

PDS followed by ACT is considered the first line standard of care for advanced epithelial ovarian cancer. The EORTC 55791 trial [42] and the CHORUS trial [43] showed a similar survival in the neo-adjuvant therapy group followed by IDS compared to primary debulking followed by ACT. A meta-analysis [44] of these studies demonstrated a significantly improved OS for stage IV ovarian cancer patients in the NACT group (24.3 versus 21.1 months, HR 0.76 (95%CI 0.58–1.00), *p* = 0.048). Peri- and post-operative morbidity and mortality occurred less frequently in the NACT group. Nowadays, NACT is selected in patients where upfront debulking surgery will not lead to complete cytoreduction or when the comorbidities of the patient indicate high peri/post-operative morbidity/mortality [4]. 

Our study results indicate that the sequence/order of surgery and chemotherapy alters the immune environment differently. NACT positively influences the immune environment, with a decrease in immunosuppression, offering a window of opportunity to introduce immunotherapy during the primary treatment of advanced ovarian cancer. This temporary beneficial window is not present when debulking surgery is performed upfront. 

The immune changes induced during first line treatment are important to understand before starting combinatorial immunotherapy trials. The JAVELIN ovarian 100 (NCT02718417), which combined carboplatin–paclitaxel with avelumab (anti-PD-L1) in previously untreated patients with epithelial ovarian cancer, was interrupted early due to significantly lower PFS in the arm with avelumab as maintenance therapy after chemotherapy (Hazard ratio (HR) of 1.43 with 95% CI of 1.051⌓1.946) [24]. The arm starting treatment with avelumab simultaneously with primary chemotherapy and continued as maintenance therapy, showed a insignificantly lower PFS compared to chemotherapy alone (HR 1.14 with 95%CI of 0.832–1.565) [24]. Several combinatorial immunotherapy trials are currently ongoing (e.g., KEYLYNK-001/ENGOT-ov43/BGOG-OV43 (NCT03740165) [45], FIRST/BGOG-ov44 (NCT03602859) [46], DUO-O/BGOG-OV46 (NCT03737643) [47] or IMagyn050/GOG 3015/ENGOT-OV39 (NCT03038100) [25]). The first results of the IMagyn050 [48] also showed no advantages of combining atezolizumab (anti-PD-L1) in a primary setting. Both JAVELIN Ovarian 100 and IMagyn050 had one therapeutic arm that administered anti-PDL1 and carboplatin–paclitaxel simultaneously from the start of treatment (i.e., at diagnosis, in the case of NACT or after debulking surgery in case of upfront surgery). From an immunological point of view, based on the results of our study, both situations are compatible, resulting in a less favourable immune microenvironment. The true benefit of chemotherapy seems to be achieved after NACT. In an earlier preclinical study, we demonstrated the importance of the sequence of treatments in an ID8-fLuc serous ovarian cancer mouse model [26], where survival in mice was altered depending on the order in which immunotherapy and chemotherapy were given.

## 4. Materials and Methods

### 4.1. Study Set Up

Ovarian cancer patients were prospectively recruited between 2015 and 2017 in University Hospitals Leuven, Belgium. The study was approved by the local ethical committee (Ethics Committee Research UZ/KU Leuven, Belgium, s56311 and s64035). All of the included patients signed an informed consent. Inclusion criteria were women who were newly diagnosed with invasive ovarian cancer. Patients were excluded in the case of a concomitant second tumour, the presence of immune disease, treatments with immunomodulators, pregnancy at diagnoses, surgical removal of the primary tumour before inclusion, infectious serology (HIV, hepatitis B or hepatitis C), and/or age below 18 years. Patients who had an infection at the moment of planned inclusion were not sampled. Since an infection also mostly implied the planned treatment had to be postponed, blood sampling was then done at the moment of postponed treatment. 

### 4.2. Serum Samples

**Figure 5:**
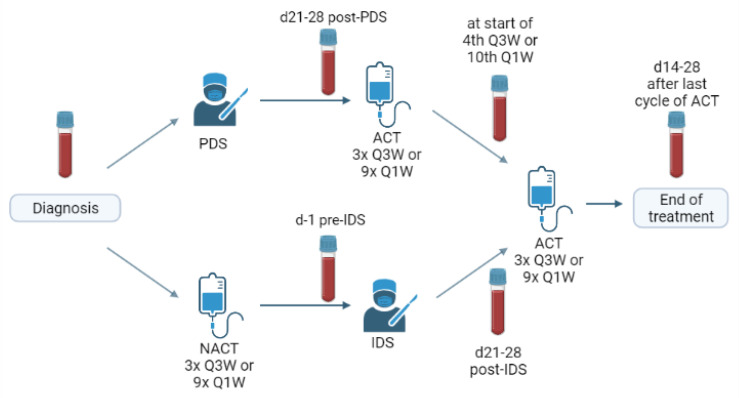
Schematic overview of treatment schedule and the timing of blood sampling. PDS: primary debulking surgery, IDS: interval debulking surgery, NACT: neoadjuvant chemotherapy, ACT: adjuvant chemotherapy, Q3W: once every three weeks, Q1W: weekly.

Depending on the preoperative assessments, patients received either upfront (debulking) surgery (PDS) followed by adjuvant chemotherapy or received neoadjuvant chemotherapy (NACT) first and then interval debulking surgery (IDS) followed by the completion of their chemotherapy [40]. Serial blood samples were taken at the different time points in the primary therapy schedule: at diagnosis, after surgery, after NACT or in the middle of the adjuvant chemotherapy and at the end of primary treatment (=two-four weeks after the last chemotherapy). In the ideal scenario, all of the patients had four consecutive blood samples (Figure 5).

The following clinical parameters were recorded: age at diagnosis, FIGO stage [3], histology, primary treatment strategy (primary debulking surgery versus NACT with interval debulking surgery), residual tumour after debulking surgery, and survival (disease specific survival (DSS), progression free survival (PFS) and overall survival (OS)).

### 4.3. Protein Analysis

Proteins were measured with a Luminex assay, according to the manufacturers’ instructions and as described earlier by our group [12]. The following immune-related proteins were analysed: interleukin-10 (IL-10), IL-12, IL-17 arginase, eotaxin-1/C-C motif chemokine ligand 11 (CCL11), eotaxin-2/C-C motif chemokine ligand 24 (CCL24), transforming growth factor β/latency-associated peptide (TGF-β/LAP), galectin-3, matrix metallopeptidase-1 (MMP-1), MMP-7, MMP-8, MMP-9, MMP-12, MMP-13, interferon gamma-induced protein 10/C-X-C motif chemokine ligand 10 (IP-10/CXCL10), macrophage-derived chemokine/C-C motif chemokine ligand 22 (MDC/CCL22), monokine induced by gamma interferon/C-X-C motif chemokine ligand 9 (MIG/CXCL9), osteopontin (OPN), vascular endothelial growth factor (VEGF), RANTES/C-C motif chemokine ligand 5 (CCL5), serum amyloid A (SAA), stromal cell-derived factor 1 alpha/C-X-C motif chemokine ligand 12 (SDF-1alpha/CXCL12).

### 4.4. Statistical Analysis

For the statistical analyses, missing values and values that were out of range were addressed using multiple imputation [28]. These imputations were based on the following variables: the proteins, age, treatment, and timepoint. The Wilcoxon signed-rank test was used to look at the effect of an intervention on the proteins (comparing the sample before and after the intervention). Results between the NACT treatment arm and the ACT treatment arm were compared with the Mann–Whitney U test. A 5% significance level was assumed for these tests. 

Univariable Cox proportional hazard models were built to assess the impact of the protein levels on the PFS. All of the statistical analyses were performed in R version 3.5.1.

## 5. Conclusions

Each step in the primary treatment schedule for ovarian cancer has its specific influence on the immune microenvironment. Based on the above results, there seems to be a window of opportunity after NACT for immunotherapy. The increase in immunostimulation and a decrease in immunosuppression creates a favourable immune environment to treat cancer, which might lead to an improved PFS in ovarian cancer. However, more information on the immune system is needed and should also be obtained during ongoing clinical trials. Our results demonstrate that this can be achieved both easily and reliably by serial measurement of immune-related proteins in blood samples.

## Figures and Tables

**Table 1:** Patient characteristics of the study population.

Characteristics	Number of Patients	%
FIGO stadium		
I	11/90	12
II	2/90	2
III	32/90	36
IV	45/90	50
Histology		
HGSOC	65/90	72
CCC	7/90	8
Endometrioid	4/90	4
LGSOC	6/90	7
Mucinous	4/90	4
Other epithelial tumours	3/90	3
No histology	1/90	1
Chemotherapy regimen		
NACT—trajectory	55/90	61
Carboplatin + paclitaxel	10/90	11
Carboplatin + paclitaxel + Bevacizumab	32/90	36
Carboplatin monotherapy	4/90	4
Other *	9/90	10
		
ACT—trajectory	30/90	33
Carboplatin + paclitaxel	23/90	26
Carboplatin + paclitaxel + Bevacizumab	3/90	3
Carboplatin monotherapy	4/90	4
		
No adjuvant chemotherapy	5/90	6
R-resection [27,28]		
R0	71/90	79
R1	2/90	2
R2	1/90	1
No surgery	16/90	18
Relapse		
Yes	54/90	60
No	36/90	40
Death		
DOD	34/90	38
AWED	24/90	27
NED	32/90	36

* Other: Taxotere and carboplatin (*n* = 2), Debio trial (NCT01930292) (*n* = 6), and paclitaxel and cisplatin started from chemo cycle six after receiving first five weekly cycles of carboplatin-paclitaxel (*n* = 1). Abbreviations: ACT, adjuvant chemotherapy; CCC, clear cell carcinoma; HGSOC, high-grade serous ovarium carcinoma; LGSOC, low-grade serous ovarium carcinoma; NACT, neo-adjuvant chemotherapy; PDS, primary debulking surgery; R0 resection, complete resection; R1 resection, optimal resection with <1 cm macroscopic tumour; R2 resection, >1 cm macroscopic tumour; DOD, death of disease; AWED, alive with evidence of disease; NED, no evidence of disease.

**Table 2:** Effect of the sequence in chemotherapy and debulking surgery on the immune environment.

Proteins	NACT Group Median	ACT Group Median	Adjusted *p*-Value
Eotaxin-1 (CCL11)	−0.3256	−0.0299	0.0900
Eotaxin-2 (CCL24)	−0.0533	0.0598	0.3702
IP-10 (CXCL10)	−0.4989	−0.7101	0.3676
MIG (CXCL9)	−0.2177	−0.3920	0.3376
SDF1a (CXCL12a)	−0.3149	−0.0857	0.3548
VEGF-A	−0.1470	−0.2192	0.5021
MMP-1	−0.2100	−0.0150	0.4619
MMP-7	−0.1282	0.2073	***0.0160***
MMP-8	0.1462	−0.0938	0.2932
Arginase	−0.4091	−0.8253	0.2908
MDC (CCL22)	0.0410	−0.5603	***0.0004***
MMP-9	−0.1882	−0.5729	0.2076
Rantes (CCL5)	0.2248	−0.4855	***0.0013***
SAA	0.0399	−0.0188	0.4688
Osteopontin	−0.0709	−0.0695	0.6585
IL-10	−0.1920	0.4215	***0.0002***

NACT group (*n* = 36): serum samples at diagnosis versus after interval debulking surgery. ACT group (*n* = 30): serum samples at diagnosis versus middle of adjuvant chemotherapy. Stage I ovarian cancer patients were excluded from this analysis. Only patients with complete resection during (primary or interval) debulking surgery were included (R0 resection). Mann–Whitney U test was applied. Abbreviations: CCL, C-C motif chemokine ligand; CXCL, C-X-C motif chemokine ligand; IL-10, interleukin-10; MMP, matrix metallopeptidase; SAA, serum amyloid A; vascular endothelial growth factor (VEGF).

## Data Availability

Data is available upon request.

## Supplementary Materials

The following are available online at https://www.mdpi.com/article/10.3390/cancers13235899/s1, Figure S1: Overview of missing data on proteins in ACT group and NACT group, Table S1: Subgroup analysis on the effect of neo-adjuvant platin-based chemotherapy, Figure S2: Effect of carboplatin-based chemotherapy without tumour load, Figure S3: Effect of debulking surgery on immune related proteins without Stage I ovarian cancers, Figure S4: Effect of chemotherapy in operated patients, Figure S5: Effect of adjuvant chemotherapy on immune-related proteins without stage I ovarian cancers, Figure S6: Effect of primary treatment on immune-related proteins without stage I ovarian cancers.

## References

[1] Bray F., Me J.F., Soerjomataram I., Siegel R.L., Torre L.A., Jemal A. (2018). Global cancer statistics 2018: GLOBOCAN estimates of incidence and mortality worldwide for 36 cancers in 185 countries. CA A Cancer J. Clin..

[2] Cancer Research UK Ovarian Cancer Survival Statistics Cancer Research UK. https://www.cancerresearchuk.org/health-professional/cancer-statistics/statistics-by-cancer-type/ovarian-cancer/survival#heading-Three.

[3] Prat J. (2014). FIGO Committee on Gynecologic Oncology Staging classification for cancer of the ovary, fallopian tube, and peritoneum. Int. J. Gynecol. Obstet..

[4] Mahmood R.D., Morgan R.D., Edmondson R.J., Clamp A.R., Jayson G.C. (2020). First-Line Management of Advanced High-Grade Serous Ovarian Cancer. Curr. Oncol. Rep..

[5] Burger R.A., Brady M.F., Bookman M.A., Fleming G.F., Monk B.J., Huang H., Mannel R.S., Homesley H.D., Fowler J., Greer B.E. (2012). Incorporation of Bevacizumab in the Primary Treatment of Ovarian Cancer. Obstet. Gynecol. Surv..

[6] Oza A., Cook A.D., Pfisterer J., Embleton-Thirsk A., Ledermann J.A., Pujade-Lauraine E., Kristensen G., Carey M.S., Beale P., Cervantes A. (2015). Standard chemotherapy with or without bevacizumab for women with newly diagnosed ovarian cancer (ICON7): Overall survival results of a phase 3 randomised trial. Lancet Oncol..

[7] Perren T., Swart A.M., Pfisterer J., Ledermann J.A., Pujade-Lauraine E., Kristensen G., Carey M.S., Beale P., Cervantes A., Kurzeder C. (2011). A Phase 3 Trial of Bevacizumab in Ovarian Cancer. N. Engl. J. Med..

[8] Moore K., Colombo N., Scambia G., Kim B.-G., Oaknin A., Friedlander M., Lisyanskaya A., Floquet A., Leary A., Sonke G. (2018). Maintenance Olaparib in Patients with Newly Diagnosed Advanced Ovarian Cancer. N. Engl. J. Med..

[9] Ray-Coquard I., Pautier P., Pignata S., Pérol D., Martín A.G., Berger R., Fujiwara K., Vergote I., Colombo N., Mäenpää J. (2019). Olaparib plus Bevacizumab as First-Line Maintenance in Ovarian Cancer. N. Engl. J. Med..

[10] Martín A.G., Pothuri B., Vergote I., Christensen R.D., Graybill W., Mirza M.R., McCormick C., Lorusso D., Hoskins P., Freyer G. (2019). Niraparib in Patients with Newly Diagnosed Advanced Ovarian Cancer. N. Engl. J. Med..

[11] Coleman R.L., Fleming G.F., Brady M.F., Swisher E.M., Steffensen K.D., Friedlander M., Okamoto A., Moore K.N., Ben-Baruch N.E., Werner T.L. (2019). Veliparib with First-Line Chemotherapy and as Maintenance Therapy in Ovarian Cancer. N. Engl. J. Med..

[12] Landolfo C., Achten E., Ceusters J., Baert T., Froyman W., Heremans R., Vanderstichele A., Thirion G., Van Hoylandt A., Claes S. (2020). Assessment of protein biomarkers for preoperative differential diagnosis between benign and malignant ovarian tumors. Gynecol. Oncol..

[13] Ledermann J.A., Raja F.A., Fotopoulou C., Gonzalez-Martin A., Colombo N., Sessa C. (2013). Newly diagnosed and relapsed epithelial ovarian carcinoma: ESMO Clinical Practice Guidelines for diagnosis, treatment and follow-up. Ann. Oncol..

[14] Van Calster B., Timmerman D., Bourne T., Testa A.C., Van Holsbeke C., Domali E., Jurkovic D., Neven P., Van Huffel S., Valentin L. (2007). Discrimination Between Benign and Malignant Adnexal Masses by Specialist Ultrasound Examination Versus Serum CA-125. J. Natl. Cancer Inst..

[15] Schreiber R.D., Old L.J., Smyth M.J. (2011). Cancer Immunoediting: Integrating Immunity’s Roles in Cancer Suppression and Promotion. Science.

[16] Curiel T.J., Coukos G., Zou L., Alvarez X., Cheng P., Mottram P., Evdemon-Hogan M., Conejo-Garcia J., Zhang L., Burow M. (2004). Specific recruitment of regulatory T cells in ovarian carcinoma fosters immune privilege and predicts reduced survival. Nat. Med..

[17] Zhang L., Conejo-Garcia J., Katsaros D., Gimotty P.A., Massobrio M., Regnani G., Makrigiannakis A., Gray H., Schlienger K., Liebman M.N. (2003). Intratumoral T Cells, Recurrence, and Survival in Epithelial Ovarian Cancer. N. Engl. J. Med..

[18] Coosemans A., Baert T., Ceusters J., Busschaert P., Landolfo C., Verschuere T., Van Rompuy A.-S., Vanderstichele A., Froyman W., Neven P. (2019). Myeloid-derived suppressor cells at diagnosis may discriminate between benign and malignant ovarian tumors. Int. J. Gynecol. Cancer.

[19] Vankerckhoven A., Wouters R., Mathivet T., Ceusters J., Baert T., Van Hoylandt A., Gerhardt H., Vergote I., Coosemans A. (2020). Opposite Macrophage Polarization in Different Subsets of Ovarian Cancer: Observation from a Pilot Study. Cells.

[20] Izar B., Tirosh I., Stover E.H., Wakiro I., Cuoco M.S., Alter I., Rodman C., Leeson R., Su M.-J., Shah P. (2020). A single-cell landscape of high-grade serous ovarian cancer. Nat. Med..

[21] Yarchoan M., Hopkins A., Jaffee E.M. (2017). Tumor Mutational Burden and Response Rate to PD-1 Inhibition. N. Engl. J. Med..

[22] Paijens S.T., Leffers N., Daemen T., Helfrich W., Boezen H.M., Cohlen B.J., Melief C.J., de Bruyn M., Nijman H.W. (2018). Antigen-specific active immunotherapy for ovarian cancer. Cochrane Database Syst. Rev..

[23] Merck and Pfizer Provide Update on on JAVELIN Ovarian 200 Trial of Avelumab in Platinum-Resistant/Refractory Ovarian Cancer. https://investors.pfizer.com/investor-news/press-release-details/2018/Merck-KGaA-Darmstadt-Germany-and-Pfizer-Provide-Update-on-Avelumab-in-Platinum-Resistant-Refractory-Ovarian-Cancer/default.aspx.

[24] Monk B.J., Colombo N., Oza A.M., Fujiwara K., Birrer M.J., Randall L., Poddubskaya E.V., Scambia G., Shparyk Y.V., Lim M.C. (2021). Chemotherapy with or without avelumab followed by avelumab maintenance versus chemotherapy alone in patients with previously untreated epithelial ovarian cancer (JAVELIN Ovarian 100): An open-label, randomised, phase 3 trial. Lancet Oncol..

[25] Moore K.N., Pignata S. (2019). Trials in progress: IMagyn050/GOG 3015/ENGOT-OV39. A Phase III, multicenter, randomized study of atezolizumab versus placebo administered in combination with paclitaxel, carboplatin, and bevacizumab to patients with newly-diagnosed stage III or stage IV ovarian, fallopian tube, or primary peritoneal cancer. Int. J. Gynecol. Cancer.

[26] Coosemans A., Vankerckhoven A., Baert T., Boon L., Ruts H., Riva M., Blagden S., Delforge M., Concin N., Mirza M. (2019). Combining conventional therapy with immunotherapy: A risky business?. Eur. J. Cancer.

[27] Du Bois A., Reuss A., Pujade-Lauraine E., Harter P., Ray-Coquard I., Pfisterer J. (2009). Role of surgical outcome as prognostic factor in advanced epithelial ovarian cancer: A combined exploratory analysis of 3 prospectively randomized phase 3 multicenter trials: By the Arbeitsgemeinschaft Gynaekologische Onkologie Studiengruppe Ovarialkarzinom (AGO-OVAR) and the Groupe d’Investigateurs Nationaux Pour les Etudes des Cancers de l’Ovaire (GINECO). Cancer.

[28] Karam A., Ledermann J.A., Kim J.-W., Sehouli J., Lu K., Gourley C., Katsumata N., Burger R.A., Nam B.-H., Bacon M. (2017). Fifth Ovarian Cancer Consensus Conference of the Gynecologic Cancer InterGroup: First-line interventions. Ann. Oncol..

[29] Coosemans A., Decoene J., Baert T., Laenen A., Kasran A., Verschuere T., Seys S., Vergote I. (2016). Immunosuppressive parameters in serum of ovarian cancer patients change during the disease course. OncoImmunology.

[30] Khairallah A.S., Genestie C., Auguste A., Leary A. (2018). Impact of neoadjuvant chemotherapy on the immune microenvironment in advanced epithelial ovarian cancer: Prognostic and therapeutic implications. Int. J. Cancer.

[31] Jiménez-Sánchez A., Memon D., Pourpe S., Veeraraghavan H., Li Y., Vargas H.A., Gill M.B., Park K.J., Zivanovic O., Konner J. (2017). Heterogeneous Tumor-Immune Microenvironments among Differentially Growing Metastases in an Ovarian Cancer Patient. Cell.

[32] Heindl A., Lan C., Rodrigues D.N., Koelble K., Yuan Y. (2016). Similarity and diversity of the tumor microenvironment in multiple metastases: Critical implications for overall and progression-free survival of high-grade serous ovarian cancer. Oncotarget.

[33] Zhang A.W., McPherson A., Milne K., Kroeger D.R., Hamilton P.T., Miranda A., Funnell T., Little N., de Souza C.P., Laan S. (2018). Interfaces of Malignant and Immunologic Clonal Dynamics in Ovarian Cancer. Cell.

[34] Baert T., Vergote I., Coosemans A. (2017). Ovarian cancer and the immune system. Gynecol. Oncol. Rep..

[35] Wu X., Feng Q.-M., Wang Y., Shi J., Ge H.-L., Di W. (2009). The immunologic aspects in advanced ovarian cancer patients treated with paclitaxel and carboplatin chemotherapy. Cancer Immunol. Immunother..

[36] Coleman S., Clayton A., Mason M.D., Jasani B., Adams M., Tabi Z. (2005). Recovery of CD8+ T-Cell Function During Systemic Chemotherapy in Advanced Ovarian Cancer. Cancer Res..

[37] Yi J.S., Ready N., Healy P., Dumbauld C., Osborne R., Berry M., Shoemaker D., Clarke J., Crawford J., Tong B. (2017). Immune Activation in Early-Stage Non–Small Cell Lung Cancer Patients Receiving Neoadjuvant Chemotherapy Plus Ipilimumab. Clin. Cancer Res..

[38] De Goeje P.L., Poncin M., Bezemer K., Kaijen-Lambers M.E., Groen H.J., Smit E.F., Dingemans A.-M.C., Kunert A., Hendriks R.W., Aerts J.G. (2019). Induction of Peripheral Effector CD8 T-cell Proliferation by Combination of Paclitaxel, Carboplatin, and Bevacizumab in Non–small Cell Lung Cancer Patients. Clin. Cancer Res..

[39] Bakos O., Lawson C., Rouleau S., Tai L.-H. (2018). Combining surgery and immunotherapy: Turning an immunosuppressive effect into a therapeutic opportunity. J. Immunother. Cancer.

[40] Wu M., Chen X., Lou J., Zhang S., Zhang X., Huang L., Sun R., Huang P., Pan S., Wang F. (2017). Changes in regulatory T cells in patients with ovarian cancer undergoing surgery: Preliminary results. Int. Immunopharmacol..

[41] Nowak M., Głowacka E., Lewkowicz P., Banasik M., Szyłło K., Zimna K., Bednarska K., Klink M. (2018). Sub-optimal primary surgery leads to unfavorable immunological changes in ovarian cancer patients. Immunobiology.

[42] Vergote I., Tropé C.G., Amant F., Kristensen G.B., Ehlen T., Johnson N., Verheijen R.H., Van Der Burg M.E., Lacave A.J., Panici P.B. (2010). Neoadjuvant Chemotherapy or Primary Surgery in Stage IIIC or IV Ovarian Cancer. N. Engl. J. Med..

[43] Kehoe S., Hook J., Nankivell M., Jayson G., Kitchener H., Lopes A.D.B., Luesley D., Perren T., Bannoo S., Mascarenhas M. (2015). Primary chemotherapy versus primary surgery for newly diagnosed advanced ovarian cancer (CHORUS): An open-label, randomised, controlled, non-inferiority trial. Lancet.

[44] Vergote I., Coens C., Nankivell M., Kristensen G.B., Parmar M.K.B., Ehlen T., Jayson G., Johnson N., Swart A.M., Verheijen R. (2018). Neoadjuvant chemotherapy versus debulking surgery in advanced tubo-ovarian cancers: Pooled analysis of individual patient data from the EORTC 55971 and CHORUS trials. Lancet Oncol..

[45] Study of Chemotherapy with Pembrolizumab (MK-3475) Followed by Maintenance with Olaparib (MK-7339) for the First-Line Treatment of Women with BRCA Non-mutated Advanced Epithelial Ovarian Cancer (EOC) (MK-7339-001/KEYLYNK-001/ENGOT-ov43/GOG-3036)—Full Text View—ClinicalTrials.gov. https://clinicaltrials.gov/ct2/show/NCT03740165.

[46] A Phase 3 Comparison of Platinum-based Therapy with TSR-042 and Niraparib Versus Standard of Care (SOC) Platinum-based Therapy as First-line Treatment of Stage III or IV Nonmucinous Epithelial Ovarian Cancer—Full Text View—ClinicalTrials.gov. https://clinicaltrials.gov/ct2/show/NCT03602859.

[47] Durvalumab Treatment in Combination with Chemotherapy and Bevacizumab, Followed by Maintenance Durvalumab, Bevacizumab and Olaparib Treatment in Advanced Ovarian Cancer Patients—Full Text View—ClinicalTrials.gov. https://clinicaltrials.gov/ct2/show/NCT03737643.

[48] Moore K., Bookman M., Sehouli J., Miller A., Anderson C., Scambia G., Myers T., Taskiran C., Robison K., Maenpaa J. (2020). LBA31 Primary results from IMagyn050/GOG 3015/ENGOT-OV39, a double-blind placebo (pbo)-controlled randomised phase III trial of bevacizumab (bev)-containing therapy +/- atezolizumab (atezo) for newly diagnosed stage III/IV ovarian cancer (OC). Ann. Oncol..

