# Supplementary material for: Neo-Adjuvant Chemotherapy Reduces, and Surgery Increases Immunosuppression in First-Line Treatment for Ovarian Cancer
Source: Cancers (Basel). 2021 Nov 24;13(23):5899. doi: 10.3390/cancers13235899 (PMC8656504; doi:10.3390/cancers13235899)
Supplement: Supplementary file 1 [file cancers-13-05899-s001.zip › cancers-1388014-supple-revised.pdf]

Supplementary figures

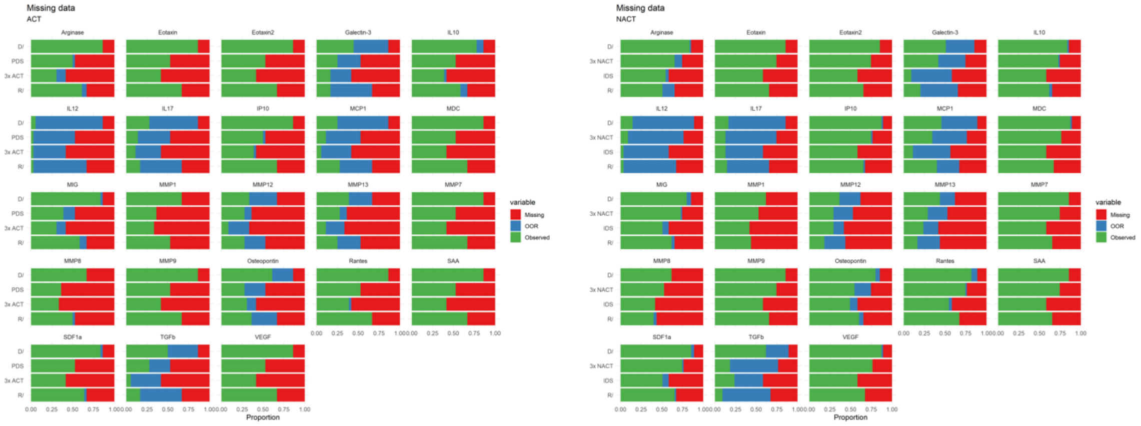

(a)

(b)

Figure S1. Overview of missing data on proteins in ACT (a) group and NACT (b) group. *Abbreviations*: CCL, C-C Motif Chemokine Ligand; IL, Interleukin; IP, Interferon gamma-Induced Protein; CXCL, C-X-C Motif Chemokine Ligand; TGF- β, Transforming Growth Factor β; LAP, Latency-Associated Peptide; MDC, Macrophage-Derived Chemokine; MIG, Monokine Induced by Gamma interferon; MMP, Matrix metallopeptidase; OPN, Osteopontin; SAA, Serum Amyloid A; SDF-1alpha, Stromal cell-Derived Factor 1 alpha; VEGF, Vascular Endothelial Growth Factor (VEGF)

Table S1. Subgroup analysis on the effect of neo-adjuvant platin-based chemotherapy

	**Platin-based chemotherapy (n = 55)**		**Carboplatin-paclitaxel +/- Bevacizumab* (n = 49)**		**Carboplatin-paclitaxel with Bevacizumab (n = 32)**		**Carboplatin-paclitaxel (n = 10)**	
	**Difference**	**p-value**	**Difference**	**p-value**	**Difference**	**p-value**	**Difference**	**p-value**
MIG (CXCL9)	-0.0666	0.978	-0.1115	0.921	-0.1245	0.952	-0.0776	0.997
IP-10 (CXCL10)	-0.2502	0.564	-0.4267	0.182	-0.3380	0.519	-0.3466	0.879
Rantes (CCL5)	0.2833	0.468	0.2695	0.5277	0.3201	0.575	-0.0325	0.997
SDF1a (CXCL12a)	-0.0206	0.909	-0.0643	0.936	0.1712	0.816	-0.6237	0.500
MDC (CCL22)	-0.0824	0.841	-0.1485	0.753	-0.0261	0.934	0.0844	0.985
Eotaxin-1 (CCL11)	0.5415	**0.013**	0.4756	**0.044**	0.5088	0.082	0.4793	0.603
Eotaxin-2 (CCL24)	0.2670	0.354	0.2572	0.420	0.2194	0.714	0.0576	0.963
VEGF-A	-0.2612	0.516	-0.3002	0.403	-0.0668	0.914	-0.2618	0.796
MMP-1	-0.5646	0.073	-0.5677	0.077	-0.6712	0.082	0.1020	0.992
MMP-7	-0.1512	0.747	-0.1934	0.703	-0.3410	0.536	0.5167	0.553
MMP-8	-0.3880	0.343	-0.4517	0.266	-0.1441	0.784	-0.7431	0.430
Arginase	-1.2780	**<0.001**	-1.2208	**<0.001**	-1.4400	**<0.001**	-0.4790	0.622
MMP-9	-0.7926	**<0.001**	-0.7337	**<0.001**	-0.6687	**0.043**	-1.2230	**0.023**
SAA	-0.8317	**0.001**	-0.7588	**0.003**	-1.0557	**0.002**	-0.1982	0.981
Osteopontin	-0.6535	**0.011**	-0.6757	**0.004**	-0.6669	**0.033**	-1.6347	**0.003**
IL-10	-0.8341	**0.001**	-0.8784	**<0.001**	-1.0197	**0.007**	-0.0638	0.996

*Carboplatin-paclitaxel +/- Bevacizumab includes the following patients of Table 1: “Carboplatin + paclitaxel” (n=10), “Carboplatin + paclitaxel + Bevacizumab” (n=32), and from the group “Other”, the "Debio trial (NCT01930292)" (n=6) and "Paclitaxel and Cisplatinum, started from chemo cycle six after receiving first five weekly cycles of Carboplatin-Paclitaxel (n=1)" (n=1).
Abbreviations: CCL, C-C Motif Chemokine Ligand; CXCL, C-X-C Motif Chemokine Ligand; IL-10, Interleukin-10; MMP, Matrix metallopeptidase; SAA, Serum Amyloid A; Vascular Endothelial Growth Factor (VEGF).

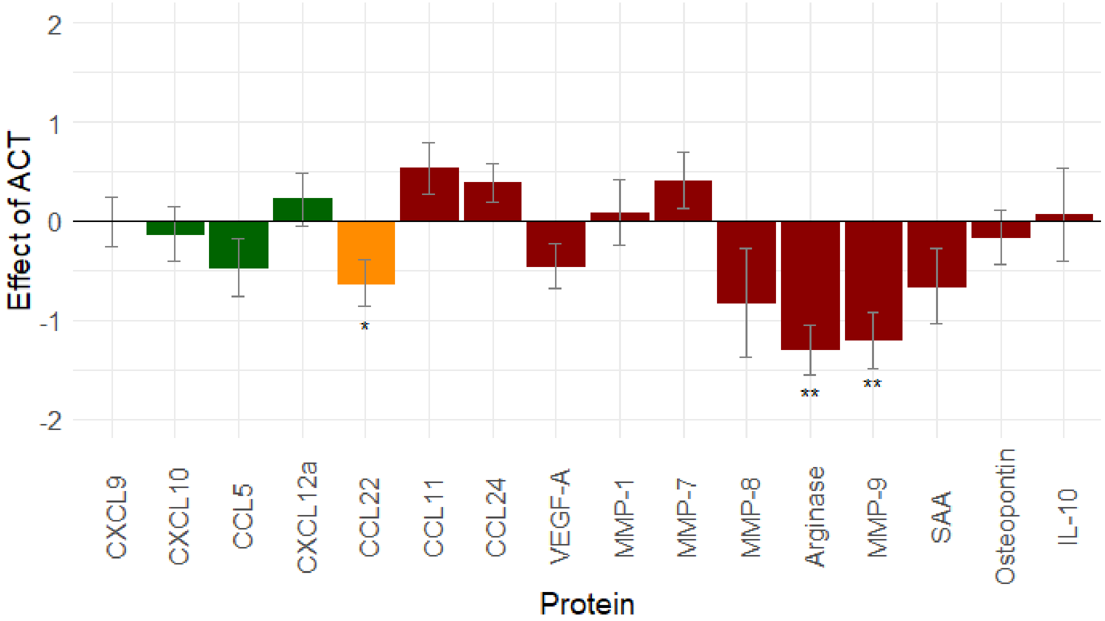

Figure S2. Effect of carboplatin-based chemotherapy without tumour load (n=30). Platin-based chemotherapy without tumour load tends to decrease immunosuppression. Only patients with complete resection during surgery (R0 resection) were included. Proteins in green are immune stimulatory factors, proteins in red are immune inhibitory factors, proteins in orange have dual function. *: 0.01 <= p-value < 0.05; **: p-value < 0.01. *Abbreviations*: ACT, adjuvant chemotherapy; CCL, C-C Motif Chemokine Ligand; CXCL, C-X-C Motif Chemokine Ligand; IL-10, Interleukin-10; MMP, Matrix metallopeptidase; SAA, Serum Amyloid A; Vascular Endothelial Growth Factor (VEGF).

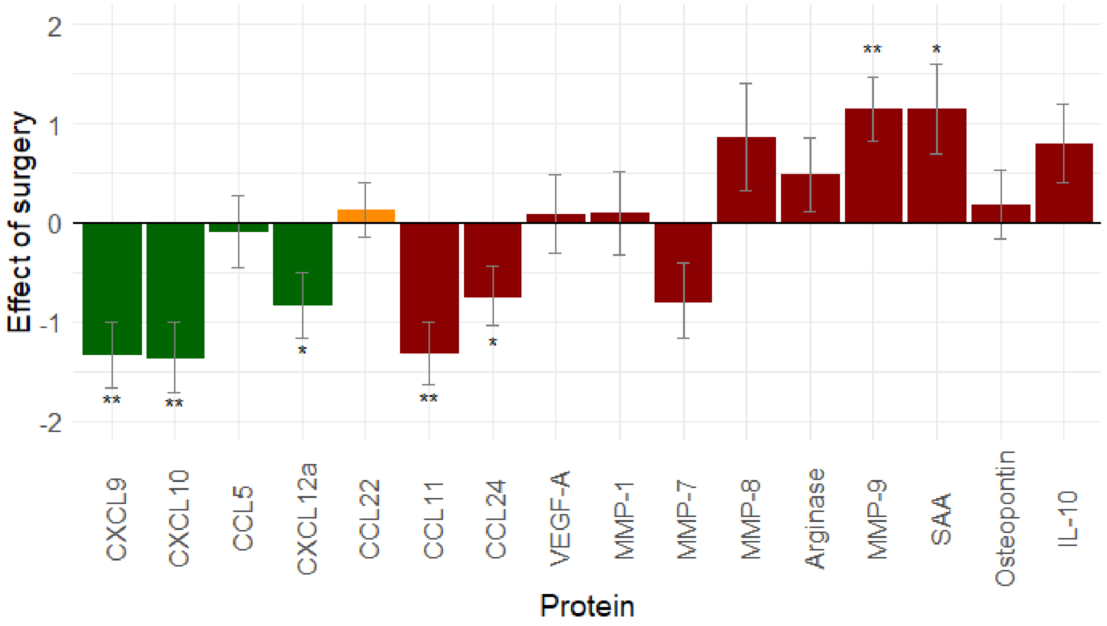

Figure S3. Effect of debulking surgery on immune related proteins without Stage I ovarian cancers (n=60). Debulking surgery tends to increase immunosuppression and decrease immunostimulation, even after excluding stage I ovarian cancers. Only patients with complete resection during surgery (R0 resection) were included. Proteins in green are immune stimulatory factors, proteins in red are immune inhibitory factors, proteins in orange have dual function. *: 0.01 <= p-value < 0.05; **: p-value < 0.01. *Abbreviations*: CCL, C-C Motif Chemokine Ligand; CXCL, C-X-C Motif Chemokine Ligand; IL-10, Interleukin-10; MMP, Matrix metallopeptidase; SAA, Serum Amyloid A; Vascular Endothelial Growth Factor (VEGF).

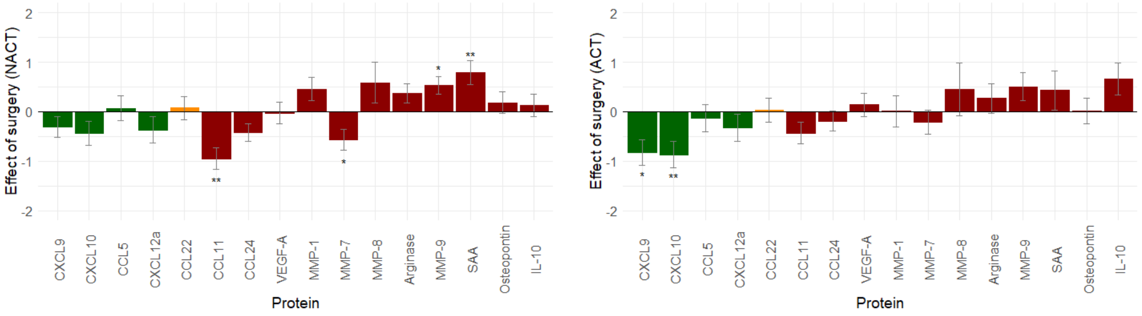

(a)

(b)

Figure S4. Effect of chemotherapy in operated patients. (a) Patients for which the difference was calculated between the sample collected after NACT and the sample taken after IDS (n=36). (b) Patients for which the difference was used between the sample at diagnosis and the sample after primary debulking surgery (PDS) (=group that received only ACT) (n=30). Surgery has a comparable effect on immune related proteins in both groups, regardless of chemotherapy pre-treatment. *: 0.01 <= p-value < 0.05;**: p-value < 0.01. *Abbreviations*: ACT, adjuvant chemotherapy; CCL, C-C Motif Chemokine Ligand; CXCL, C-X-C Motif Chemokine Ligand; IL-10, Interleukin-10; MMP, Matrix metallopeptidase; NACT, neo-adjuvant chemotherapy group; SAA, Serum Amyloid A; Vascular Endothelial Growth Factor (VEGF).

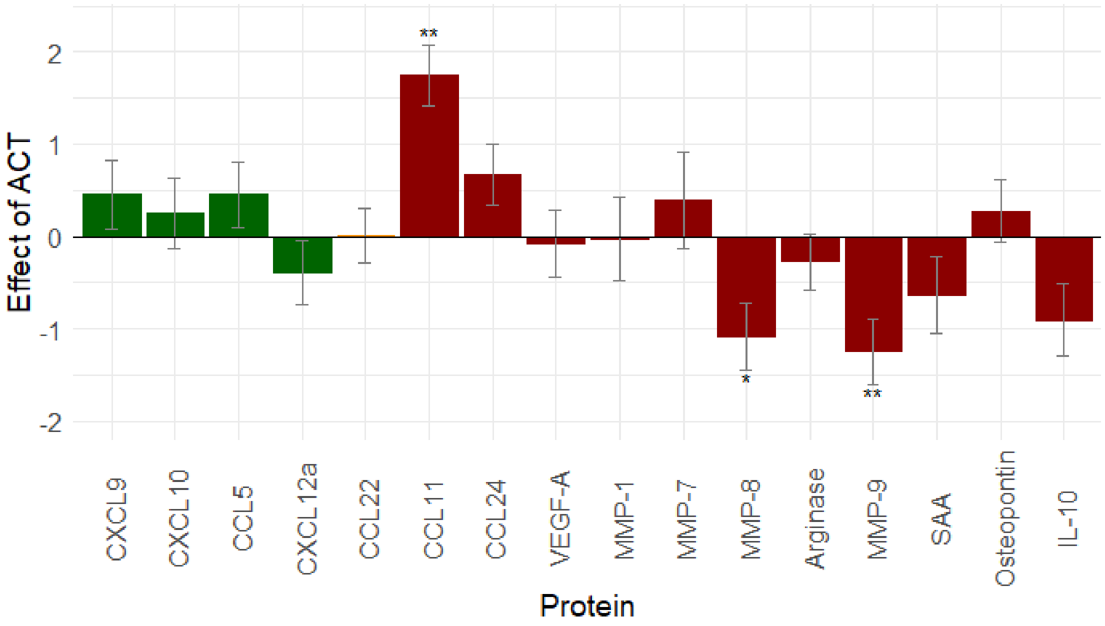

Figure S5. Effect of adjuvant chemotherapy on immune related proteins without stage I ovarian cancers (n=60). Adjuvant chemotherapy shows non-significant increase in immunostimulation and mixed effect on immunosuppression, even after excluding stage I ovarian cancer. Proteins in green are immune stimulatory factors, proteins in red are immune inhibitory factors, proteins in orange have a dual function. *: 0.01 <= p-value < 0.05; **: p-value < 0.01. Abbreviations: ACT, adjuvant chemotherapy; CCL, C-C Motif Chemokine Ligand; CXCL, C-X-C Motif Chemokine Ligand; IL-10, Interleukin-10; MMP, Matrix metallopeptidase; ACT, adjuvant chemotherapy; SAA, Serum Amyloid A; Vascular Endothelial Growth Factor (VEGF).

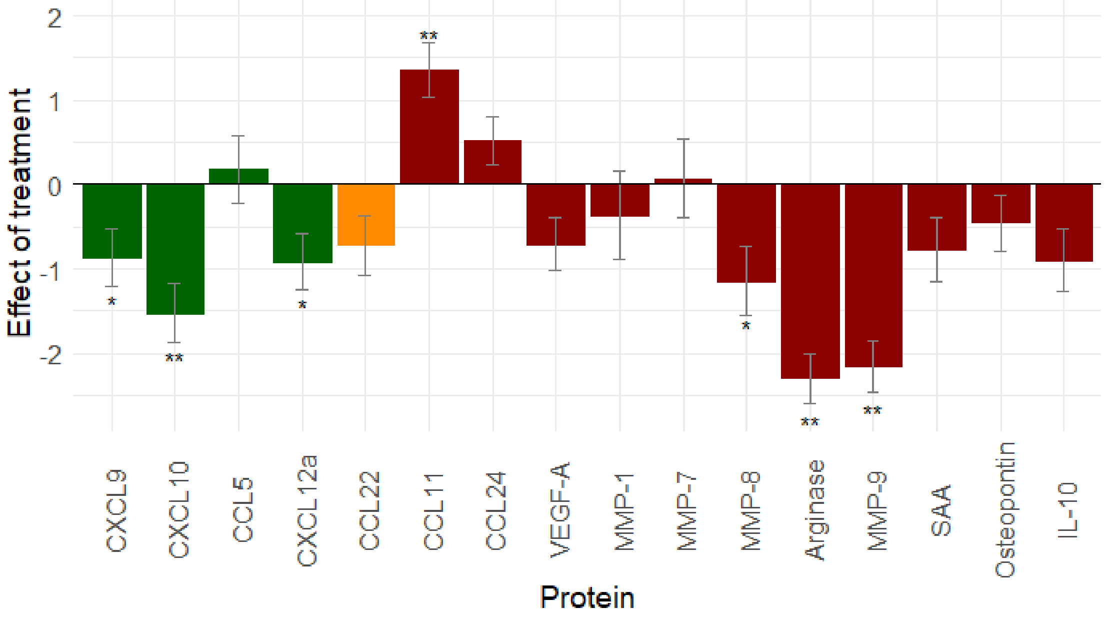

Figure S6. Effect of primary treatment on immune related proteins without stage I ovarian cancers (n=60). Primary treatment of ovarian cancer gives a pronounced decrease in immunostimulation and immunosuppression, even after excluding stage I ovarian cancer. Proteins in green are immune stimulatory factors, proteins in red are immune inhibitory factors, proteins in orange have dual function. *: 0.01 <= p-value < 0.05; **: p-value < 0.01. Abbreviations: CCL, C-C Motif Chemokine Ligand; CXCL, C-X-C Motif Chemokine

Ligand; IL-10, Interleukin-10; MMP, Matrix metallopeptidase; SAA, Serum Amyloid A; Vascular Endothelial Growth Factor (VEGF).
